# Empathy and tolerance of ambiguity in medical students and doctors participating in art-based observational training at the Rijksmuseum in Amsterdam, the Netherlands: a before-and-after study

**DOI:** 10.3352/jeehp.2025.22.3

**Published:** 2025-01-14

**Authors:** Stella Anna Bult, Thomas van Gulik

**Affiliations:** Department of Surgery, Amsterdam University Medical Center, University of Amsterdam, Amsterdam, The Netherlands; The Catholic University of Korea, Korea

**Keywords:** Cohort studies, Empathy, Medical education, Medical students, Surveys and questionnaires, Netherlands

## Abstract

**Purpose:**

This research presents an experimental study using validated questionnaires to quantitatively assess the outcomes of art-based observational training in medical students, residents, and specialists. The study tested the hypothesis that art-based observational training would lead to measurable effects on judgement skills (tolerance of ambiguity) and empathy in medical students and doctors.

**Methods:**

An experimental cohort study with pre- and post-intervention assessments was conducted using validated questionnaires and qualitative evaluation forms to examine the outcomes of art-based observational training in medical students and doctors. Between December 2023 and June 2024, 15 art courses were conducted in the Rijksmuseum in Amsterdam. Participants were assessed on empathy using the Jefferson Scale of Empathy (JSE) and tolerance of ambiguity using the Tolerance of Ambiguity in Medical Students and Doctors (TAMSAD) scale.

**Results:**

In total, 91 participants were included; 29 participants completed the JSE and 62 completed the TAMSAD scales. The results showed statistically significant post-test increases for mean JSE and TAMSAD scores (3.71 points for the JSE, ranging from 20 to 140, and 1.86 points for the TAMSAD, ranging from 0 to 100). The qualitative findings were predominantly positive.

**Conclusion:**

The results suggest that incorporating art-based observational training in medical education improves empathy and tolerance of ambiguity. This study highlights the importance of art-based observational training in medical education in the professional development of medical students and doctors.

## Graphical abstract

[Fig f5-jeehp-22-03]

## Introduction

### Background

Research has shown that there is a correlation between medical students’ exposure to the humanities and a higher level of positive personal attributes such as emotional appraisal, empathy and tolerance of ambiguity, and lower levels of negative attributes such as signs of burnout [1]. In particular, higher levels of empathy may lead to better clinical outcomes. Intolerance of ambiguity has been shown to correlate with increased psychosocial distress and poorer clinical performance [2]. These findings underscore the importance of these skills for healthcare professionals and the need to promote them in medical education.

Worldwide, there are several programs involving collaboration between medical faculties and art museums. In the Netherlands, several art-related educational programs have been developed in all medical schools, varying in duration, design, and content [3].

The Amsterdam Universitair Medisch Center (AUMC) has developed an art-based observational training program for medical students and residents, in collaboration with the Rijksmuseum in Amsterdam [4,5]. The program consists of a structured course at the Rijksmuseum by an art educator and a clinical specialist. We investigated whether this form of art-based education improves empathy and judgement skills (tolerance of ambiguity) in participants.

### Objectives

The aim of this study was to evaluate the effects of art-based observational training on medical students, residents, and specialists using quantitative methods to assess the competencies of empathy and tolerance of ambiguity. The hypothesis underlying this study was that art-based observational training would have a positive effect on these competencies.

## Methods

### Ethics statement

Informed consent was obtained from the participants. This was not a clinical trial involving a health risk; therefore, institutional review board approval was not required in the Netherlands.

### Study design

An experimental, single-group, pre- and post-test design was devised. Validated questionnaires were used to examine the outcomes of art-based observational training in medical students, residents, and specialists. The study was described according to the Transparent Reporting of Evaluations with Nonrandomized Designs (TREND) statement available at: https://www.cdc.gov/trendstatement/index.html.

### Setting

Between December 2023 and June 2024, 15 two-hour courses were held at the Rijksmuseum in Amsterdam, the Netherlands (Supplement 1). Follow-up took place after the sessions were completed. The study’s design did not include a control group and focused only on the participating cohorts.

### Participants

Participants were recruited voluntarily through the university faculty and the network of the AUMC. The inclusion criteria required that the participants were medical students, doctors, or other health professionals with regular patient contact. All participants had to be proficient in Dutch, and all were exposed to the same, 1-time intervention.

Due to the limited number of participants, it was decided not to exclude the results of the participants with missing responses in either the pre- or post-test. The corresponding question was not included in the analysis.

### Interventions

Groups consisted of 6 to 8 participants. Each group was led by an experienced clinician (T.v.G.) trained in visual thinking strategies, by the Foundation of VTS Netherlands (Stichting VTS Nederland), and a medical doctor who was studying for a master’s degree in History of Art (S.A.B.). VTS (or parts of the VTS program) were used in the structured sessions. The group discussions were adapted to the participants’ medical background [4]. The artworks had different tasks, but all were selected on the basis of an assumed physical abnormality or otherwise medical feature. The 4-stage ABCD method of art-observation was used, as previously reported (Table 1) [4,5].

### Outcomes

Pre- and post-intervention tests were administered using validated and standardized scales. The pre-intervention test had to be completed online by the participants before the museum visit and the post-intervention test was administered during the post-intervention evaluation.

### Measurement

Empathy and tolerance of ambiguity were assessed using the Jefferson Scale of Empathy and the Tolerance of Ambiguity in Medical Students and Doctors scale (TAMSAD), respectively [6,7]. The JSE was used in this study with permission from the Thomas Jefferson University. The TAMSAD is in the public domain. The JSE is a 20-item scale designed to measure empathy in health professionals [6] (Supplement 2). Items are scored on a 7-point Likert scale assessing 3 components of empathy—namely, perspective taking, compassionate care, and the ability to put oneself in the patients’ shoes. The JSE is scored between 20 and 140 points, with higher scores indicating higher levels of empathy [6].

The TAMSAD contains 29 items measuring tolerance of ambiguity in medical students and doctors [7]. Most of the items in the questionnaire refer to clinical contexts (Supplement 3). The items are answered on a 5-point Likert scale. The TAMSAD is scored from 29 to 145 points, with higher scores indicating greater tolerance of ambiguity. This score is calculated as a mean score out of 5 across the 29 items and then transformed to a score of 0–100 score using the formula: new score=25(old score–1) [7]. In both questionnaires, half of the items are negatively worded, for which the scores are reversed.

In addition to the standardized questionnaires, participants were asked to complete a qualitative evaluation form with 9 questions that could be answered on a 5-point Likert scale, with the possibility to elaborate on the answer (Supplement 4).

### Bias

Participants were recruited voluntarily, which may introduce selection bias and confirmation bias. This is considered in our discussion.

### Study size

Post-hoc power analysis was conducted to evaluate the difference between 2 dependent means (matched pairs) for the total scores of the JSE and the TAMSAD scale using G*Power (Heinrich-Heine-Universität Düsseldorf). The calculated power (1-β error probability) was 0.851 for the JSE and 0.893 for the TAMSAD. The analysis used the following parameters: a one-tailed test, effect size of 0.550 for the JSE and 0.342 for the TAMSAD, and total sample sizes of 29 (JSE) and 62 (TAMSAD). The effect size for the JSE was derived from the mean and standard deviation of the pre-test group (110.83±12.56) and the post-test group (114.53±12.04), with a correlation coefficient of 0.851 between the paired groups. Similarly, the effect size for the TAMSAD was calculated based on the pre-test mean and standard deviation (100.62±8.99) and post-test values (102.71±9.99), with a correlation coefficient of 0.798 between the groups. These parameters demonstrated robust statistical power for detecting differences in both scales, ensuring the reliability of the findings.

### Assignment method

Participants were recruited among students of the medical faculty of the University of Amsterdam and the network of the AUMC and assigned to the museum for a planned course. The first group received the JSE, and the second group completed the TAMSAD scale.

### Blinding

A blinded group was not possible in this study, nor was a control group included.

### Unit of analysis

The unit of analysis was each group of participants who took 1 of 2 surveys.

### Statistical methods

Statistical analyses of the collected data were performed using IBM SPSS Statistics ver. 29.0 for Windows (IBM Corp.). SPSS was used to analyze the pre- and post-tests for both the JSE and TAMSAD using the paired t-test. Significance was defined as P<0.05, and results were rounded to 2 decimal places.

## Results

### Participants

In total, 92 medical students, residents and specialists participated in the art-based program at the Rijksmuseum, with 1 participant being excluded. This resulted in 91 participants being included in the analysis: 63 women and 28 men. The participants represented various levels of medical training and career stages.

Thirty participants were included in the JSE group; 1 participant did not complete the post-test and was excluded from this study. Twenty-nine participants completed the JSE, and 1 participant had a missing response on the pre-test. Sixty-two participants completed the TAMSAD. Six participants had a missing response on either the pre- or post-test. The characteristics of the participants are presented in Table 2.

### Main results

**Fig. 1.**
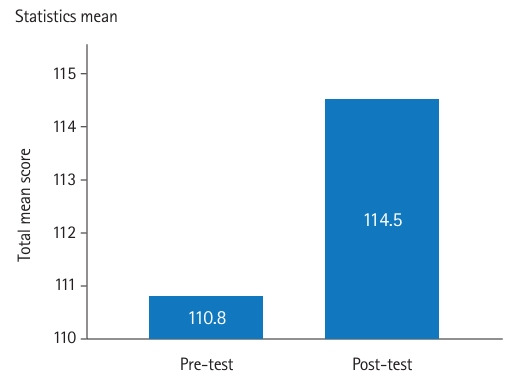
Pre- and post-intervention test results of the Jefferson Scale of Empathy (rescaled).

**Fig. 2.**
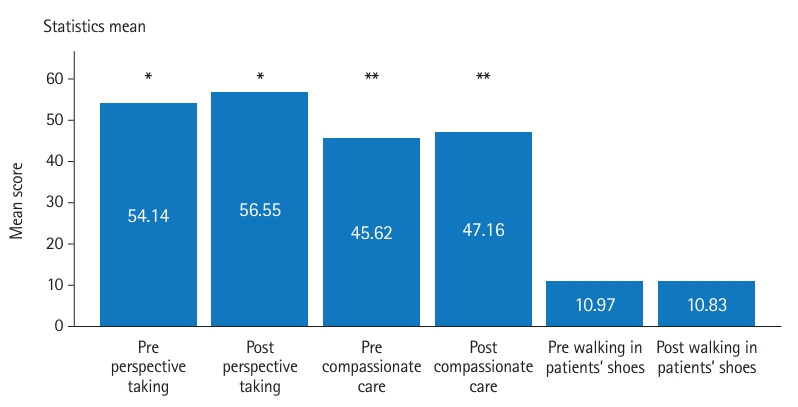
Pre- and post-intervention test results for the subscales of the Jefferson Scale of Empathy.

**Fig. 3.**
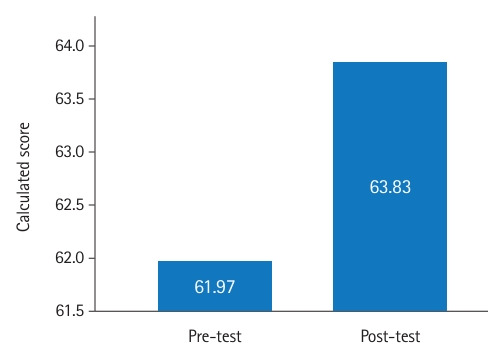
Mean differences in calculated scores between pre- and post-intervention test results of the Tolerance of Ambiguity in Medical Students and Doctors scale (rescaled).

Overall, the post-tests showed significant increases in mean and total scores for both the JSE and TAMSAD (Figs. 1–3). There were no statistically significant differences in total scores by gender, medical speciality, or function.

### Qualitative analyses; participants’ reflections on the study

**Fig. 4.**
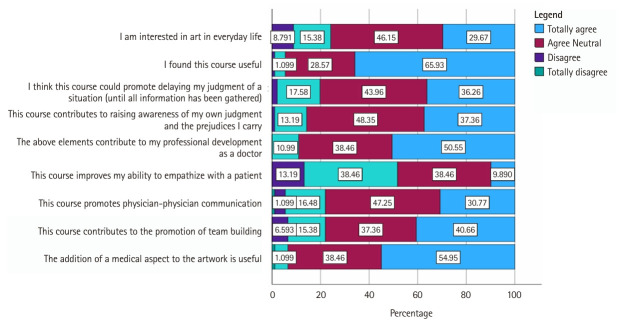
The ABCD method involves 4 stages of observing an artwork [4].

The response rate to the online evaluation form was 100%, and all 91 participants completed the questionnaire. Overall, the participants reported positive reactions towards the art-based program (Fig. 4). Some of the participants’ responses are highlighted below:

“It was a very nice, special and original approach, which allowed me to gain useful tools for my work as a doctor in psychiatry.”
‘‘I usually go straight for the symptoms and do not pay much attention to the observations.”
“You learn to observe differently and better. Good for our profession as doctors and therefore good for the patient.”

## Discussion

### Key results

Our study, which was conducted among medical students, residents, and specialists, showed that the art-based observational training in the Rijksmuseum in Amsterdam significantly increased empathy and tolerance of ambiguity. The mean difference between the pre- and post-intervention tests was 3.71 points for empathy, ranging from 20 to 140, and 1.86 points for tolerance of ambiguity, ranging from 0 to 100. We did not observe any significant differences in the results between participants in terms of gender, medical speciality, or function.

Our qualitative analysis showed positive responses; most participants reported that the art-based observational training program was useful in terms of medical education and professional development. The majority of the participants felt that the course helped them to become more aware of their own judgements and biases, and to improve their ability to delay judgement in healthcare situations. In addition, many participants found that the course improved their ability to empathize with patients, while at the same time promoting communication and teambuilding.

### Interpretation

This experimental study lends support to the view that the inclusion of art-based observational training in medical education may be valuable. Although the results showed slight increases in both empathy and tolerance of ambiguity, the results were statistically significant. This small difference may be explained by the fact that there was a single intervention that lasted for only 2 hours, providing only brief exposure to the art-based observational training program.

### Comparison with previous studies

Several studies investigating empathy with the use of the JSE in art-based education have found no significant changes, while some studies found differences in subcomponents of empathy [8]. We found a significant increase in the total score, as well as increases in the perspective taking and compassionate care subscales. A possible explanation for these differences is that the sample size for testing empathy was relatively small. In addition, our course was designed differently from previous research, which may have led to different results.

In terms of tolerance of ambiguity, most studies have used Geller’s Modified Tolerance for Ambiguity Scale [9-11]. Some of these studies found significance, while others did not. More recently, the TAMSAD has been developed. Although evidence of validity has been reported in 3 studies [12], Stephens et al. [13] found the evidence for validity to be weak but growing. As our study is the first to use the TAMSAD in this application, we cannot compare our results with previous research. Nonetheless, our study’s results are similar to those obtained using Geller’s scale [9].

The findings from our qualitative analysis align with previous research [9,14], in which the participants reflected positively on the art-based program.

### Limitations

First, we did not use a specific scale to quantify whether art-based education would have a positive effect on various situations in healthcare-related skills of medical students and doctors. We decided to focus on 2 parameters that have been used in literature on art-based observational training: empathy and tolerance of ambiguity. We chose these 2 competencies because they have been shown to improve after art-based education using standardized and validated questionnaires. Another skill that has been often described in the literature is observational skills. Our course aimed to improve observational skills, but as there are no validated scales or other methods to quantify this outcome, we chose to include it in our qualitative questionnaire, although the latter is subjective to several factors. The responses may have been influenced by participants remembering previous answers.

In particular, most of the participants were female (63 women and 28 men), which is in line with the female predominance in medical training and specialization in the Netherlands. In addition, only 29 participants were included in the group in which empathy was tested. One participant was excluded, meaning the predetermined target of 30 participants could not be reached.

As the participants were recruited voluntarily, there was selection bias. Most of the participants (75.8%), had an affinity with art before joining the program. People who regularly visited museums were more likely to be enthusiastic and to enroll in this program. Another possible limitation of this study is that the participants’ functions and levels of training were different; some were bachelor students, and others were already professionals. However, no statistically significant differences were found between functions.

Because we did not include a control group, we could not test our results against a group that did not receive art-based education.

Finally, the participants were only exposed to a single 2-hour intervention. The effects of the intervention were assessed immediately after the session. The results therefore show the short-term effects of a short intervention.

### Generalizability

The 2 measurement tools used in this study allowed us to draw conclusions about both empathy and tolerance of ambiguity in both medical students and doctors. The quantitative results confirmed the qualitative responses, adding to the strength of this study. Our art-based program could be used in other medical universities and local museums, anticipating similar outcomes.

### Suggestions

Future research should increase the number of interventions and follow-up participants over a longer period of time, to assess long-term effects. The inclusion of a control group not exposed to the program would increase the strength of the study.

Random allocation of participants would result in an unbiased group population. In order to increase the number of participants in order to make a stronger claim about the effects of art-based observational training in the Netherlands, it is recommended that the programs of the various faculties and museum collaborations be expanded and coordinated, for which the Rijksmuseum program can be used as a model.

The ultimate aim, albeit remote at present, is to include art-based observational training in all the curricula of all medical faculties in the Netherlands, ideally, at the beginning of the master’s program, before the start of clinical internships. In this way, students will be trained at an early stage to approach patients in an open-minded and empathic way.

### Conclusion

We evaluated art-based observational training among Dutch medical students, residents, and specialists in the Rijksmuseum in Amsterdam. Two measurement tools, the JSE and the TAMSAD, were used in pre- and post-intervention tests. Our results showed small but statistically significant increases in both empathy and tolerance of ambiguity after the intervention. In addition to the quantitative analyses, the qualitative evaluations also showed positive results as well. Participants were enthusiastic and found the course meaningful and a welcome addition to their regular medical training, where they are constantly challenged to observe, think, and communicate unambiguously. These findings suggest that incorporating art-based observational training into medical education has a beneficial effect on empathy and tolerance of ambiguity.

## Figures and Tables

**Figure f5-jeehp-22-03:**
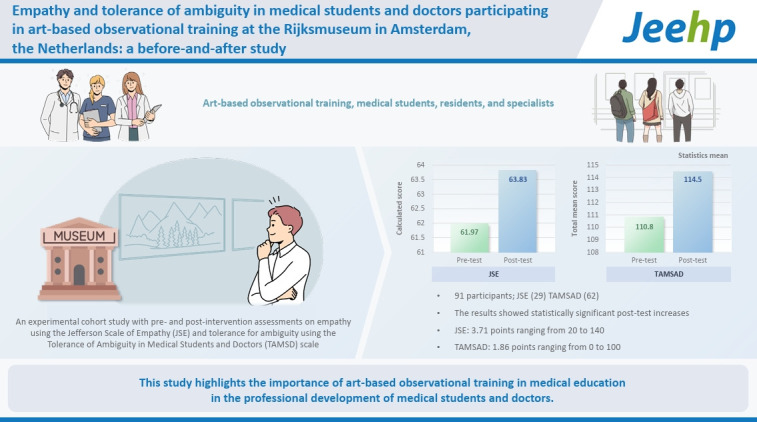


**Table 1.** The ABCD method consists of 4 stages of observing an artwork [4]

	ABCD method
A	Attention: Take your time and concentrate by looking at the object for one minute.
B	Behold: Name the objects that stand out, describe how the objects are inter-connected and find an interpretation of the work.
C	Communication: Connect with one another and discuss thoughts and experiences.
D	Diagnosis: Find a physical abnormality depicted in the work.

**Table 2.** Participants’ characteristics

Characteristic	Study population (N=91)	JSE (N=29)	TAMSAD (N=62)
Gender			
Male	28 (30.8)	9 (31.0)	19 (30.6)
Female	63 (69.2)	20 (69.0)	43 (69.4)
Function			
BS	10 (11.0)	2 (6.0)	8 (12.9)
MS	15 (16.5)	7 (24.1)	8 (12.9)
Resident	33 (36.3)	12 (41.4)	21 (33.9)
MD/PhD student	20 (22.0)	5 (17.2)	15 (24.2)
Specialist	11 (12.1)	3 (10.3)	8 (12.9)
Other	2 (2.2)		2 (3.2)
Field of medicine			
Student	25 (27.5)	9 (31.0)	16 (25.8)
Internal medicine	6 (6.6)	2 (6.9)	4 (6.5)
Surgery	15 (16.5)	10 (34.5)	5 (8.1)
Psychiatry	5 (5.5)	2 (6.9)	3 (4.8)
Gynecology	4 (4.4)		4 (6.5)
General practitioner	5 (5.5)	2 (6.9)	3 (4.8)
Anesthesiology	4 (4.4)	1 (3.4)	3 (4.8)
Urology	2 (2.2)		2 (3.2)
Geriatrics	2 (2.2)	1 (3.4)	1 (1.6)
Plastic surgery	1 (1.1)	1 (3.4)	
Pediatrics	2 (2.2)		2 (3.2)
Orthopedics	2 (2.2)	1 (3.4)	1 (1.6)
Cardiology	1 (1.1)		1 (1.6)
Global health and tropical medicine	1 (1.1)		1 (1.6)
Dentist	1 (1.1)		1 (1.6)
Emergency medicine	1 (1.1)		1 (1.6)
Radiotherapy	10 (11.0)		10 (16.1)
Dermatology	1 (1.1)		1 (1.6)
Doctor-teacher	1 (1.1)		1 (1.6)
Ear, nose, and throat specialist	1 (1.1)		1 (1.6)
Youth public health	1 (1.1)		1 (1.6)

Values are presented as number (%). JSE, Jefferson Scale of Empathy; TAMSAD, Tolerance of Ambiguity in Medical Students and Doctors; BS, Bachelor of Science; MS, Master of Science; MD, Doctor of Medicine; PhD, Doctor of Philosophy.

## Supplementary materials

Supplementary files are available from Harvard Dataverse: https://doi.org/10.7910/DVN/KRWWPT

Supplement 1. Detailed program content at the Rijksmuseum in Amsterdam.

Supplement 2. Jefferson Scale of Empathy.

Supplement 3. Tolerance of Ambiguity in Medical Students and Doctors scale.

Supplement 4. Qualitative evaluation form with open-ended responses.

Supplement 5. Audio recording of the abstract.

## References

[1] Mangione S, Chakraborti C, Staltari G, Harrison R, Tunkel AR, Liou KT, Cerceo E, Voeller M, Bedwell WL, Fletcher K, Kahn MJ (2018). Medical students’ exposure to the humanities correlates with positive personal qualities and reduced burnout: a multi-institutional U.S. survey. J Gen Intern Med.

[2] Geller G, Grbic D, Andolsek KM, Caulfield M, Roskovensky L (2021). Tolerance for ambiguity among medical students: patterns of change during medical school and their implications for professional development. Acad Med.

[3] Reijntjes M, de Ruiter P, Linsen L, Derickx M, Jansen FW, van Noort S, Kamstra S, van Gulik T, Linthorst G (2021). The place of art observation in the medical curriculum in the Netherlands. TSG.

[4] van Gulik T, Bult S, de Ruiter P (2024). Evaluation of the ABCD-method in art-based observational training of medical students and surgical residents in the museum. Int J Surg Educ [Internet].

[5] de Ruiter PE, van de Brug B, Reijntjes MA, Linsen L, Lagarde S, van Gulik TM (2024). Art-based observational training for medical students and surgical residents in two Dutch museums. Clin Ter.

[6] Hojat M, DeSantis J, Shannon SC, Mortensen LH, Speicher MR, Bragan L, LaNoue M, Calabrese LH (2018). The Jefferson Scale of Empathy: a nationwide study of measurement properties, underlying components, latent variable structure, and national norms in medical students. Adv Health Sci Educ Theory Pract.

[7] Hancock J, Roberts M, Monrouxe L, Mattick K (2015). Medical student and junior doctors’ tolerance of ambiguity: development of a new scale. Adv Health Sci Educ Theory Pract.

[8] Ike JD, Howell J (2022). Quantitative metrics and psychometric scales in the visual art and medical education literature: a narrative review. Med Educ Online.

[9] Strohbehn GW, Hoffman SJ, Tokaz M, Houchens N, Slavin R, Winter S, Quinn M, Ratz D, Saint S, Chopra V, Howell JD (2020). Visual arts in the clinical clerkship: a pilot cluster-randomized, controlled trial. BMC Med Educ.

[10] Klugman CM, Beckmann-Mendez D (2015). One thousand words: evaluating an interdisciplinary art education program. J Nurs Educ.

[11] Gowda D, Dubroff R, Willieme A, Swan-Sein A, Capello C (2018). Art as sanctuary: a four-year mixed-methods evaluation of a visual art course addressing uncertainty through reflection. Acad Med.

[12] Ndoja S, Chahine S, Saklofske DH, Lanting B (2020). The erosion of ambiguity tolerance and sustainment of perfectionism in undergraduate medical training: results from multiple samplings of a single cohort. BMC Med Educ.

[13] Stephens GC, Lazarus MD, Sarkar M, Karim MN, Wilson AB (2023). Identifying validity evidence for uncertainty tolerance scales: a systematic review. Med Educ.

[14] Zazulak J, Halgren C, Tan M, Grierson LE (2015). The impact of an arts-based programme on the affective and cognitive components of empathic development. Med Humanit.

