# Supplementary material for: Empathy and tolerance of ambiguity in medical students and doctors participating in art-based observational training at the Rijksmuseum in Amsterdam, the Netherlands: a before-and-after study
Source: J Educ Eval Health Prof. 2025 Jan 14;22:3. doi: 10.3352/jeehp.2025.22.3 (PMC11880821; doi:10.3352/jeehp.2025.22.3)
Supplement: Supplementary file 3 — Supplement 1. Detailed program content at the Rijksmuseum in Amsterdam. [file jeehp-22-03-suppl1.docx]

Supplement 1: precise program content as described in:

[1] T. van Gulik, S. Bult, and P. de Ruiter, ‘Evaluation of the ABCD-method in art-based observational training of medical students and surgical residents in the museum.’, *Int. J. Surg. Educ.*, Jun. 2024, Accessed: Jul. 31, 2024. [Online]. Available: https://www.ijsed.com/article/120058

**Program design**

Our program ‘See better, by looking at art’ was devised through collaboration of the surgical department of the Amsterdam University Medical Center and the art educational department of the Rijksmuseum in Amsterdam. The Rijksmuseum is one of the top museums in the Netherlands, famous for its unique collection of art works, spanning periods from the Middle Ages to modern and contemporary art. Medical students and surgical residents enrolled in an interactive workshop focused on art observation facilitated by an art-educator and a surgeon (TvG) who guided the group as tutors. The learning objectives have been reported previously. The program was evaluated in a post-workshop survey focusing on valuation and contribution of the course to professional development. The survey comprised a questionnaire consisting of ‘open’ questions regarding various aspects and learning objectives of the program as previously described.


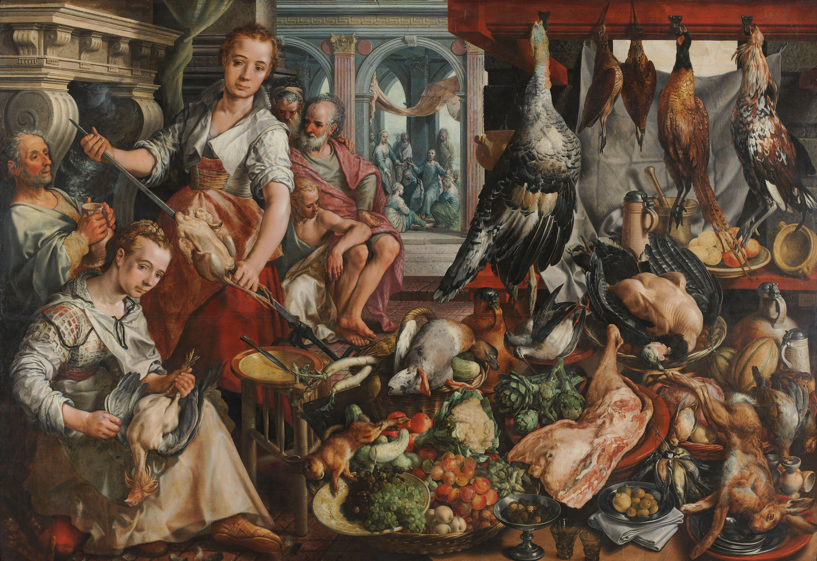

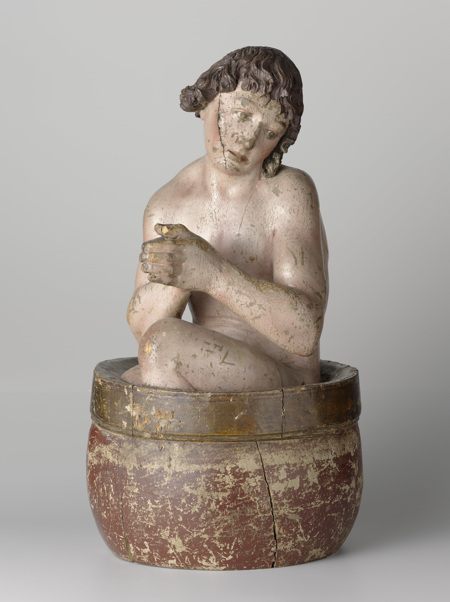


Fig. 2: Saint Vitus, anonymous, c. 1500, fruitwood, h c.58cm, Rijksmuseum Amsterdam.

Fig. 1: The Well-stocked Kitchen, with Jesus in the House of Martha and Mary in the Background, Joachim Bueckelaer, 1566, oil on panel, h 171cm × w 250cm × w 54.5kg, Rijksmuseum Amsterdam.

**The ABCD-method**

The ABCD-method refers to four stages in viewing an artwork, set on the basis of ‘observing-in-layers’ and a group discussion (Table 1). A stands for ‘attention’ to start out; ‘take your time’ and concentrate on what you see, spend one minute on carefully looking at the image. The B stands for ‘behold’, the next part of looking at an object; name the objects that stand out, describe how the objects are inter-connected, discover the context of the composition and try to come to an interpretation of the work as conveyed by the artist. The C is of ‘communication’, the way we connect with each other in exchanging thoughts generated by what we have seen and in sharing what is experienced in the group. Finally, the D stands for ‘diagnosis’ and draws attention to a medical aspect shown, that can be associated with an abnormal physical feature of a person depicted or with medical concepts at the time of the work. This is what could be called the ‘iconodiagnostic’ part of the session, associating iconographical information with a medical condition.

Table 1. The ABCD-method consists of four stages in observing an artwork

A - Attention; take your time and concentrate by looking at the object for one minute.

B - Behold; name the objects that stand out, describe how the objects are inter-connected and find an interpretation of the work

C - Communication; connect with one another and discuss thoughts and experiences.

D - Diagnosis; find a physical abnormality depicted in the work

**Content of workshops and participants**

Workshops consisted of a 2-hour tour through the museum passing by 8-10 works of art. The groups consisted of 6-12 participants of different levels of education and experience, mainly medical students, residents and doctors. Participants were engaged in exercises during the workshop to train their observational skills.8 At the end of the tour, the group would come together for evaluation and completion of the survey.

Two groups of participants were considered: In the first group (group I) participants enrolled from August 2021 to May 2023 (with a 1-year break in between because of COVID-19 restrictions). This group followed the initial structure of the art-based medical education program developed in collaboration with the Rijksmuseum, focusing on observational and communicational skills, corresponding with the A, B and C of the ABCD-method. As per recommendation of the first group, a medical aspect was included in the following workshops. Hence, from December 2023 to March 2024, all participants received the complete ABCD-method, comprising group II.

**Selection of art works**

Paintings or sculptures were selected from the vast collection of the Rijksmuseum on permanent display. A choice was made by the authors and art-educators of the Rijksmuseum on the basis of visual suitability for a group, art-historical content and a medical feature depicted. Associations with medical conditions or abnormalities were confirmed by consensus among various specialists in specific fields; general surgery, plastic surgery, orthopaedic surgery, internal medicine, neurology, psychiatry, dermatology, paediatrics, gynaecology and obstetrics.

**Exercises**

Three sorts of exercises contained in the program, were undertaken by the participants in both groups I and II.

Drawing from memory

This exercise included observing a painting for one minute after which participants were asked to turn around and draw the picture they had seen from memory on a sheet of paper. The sketches were discussed in the group, emphasizing the main points reproduced from the painting and the side points. How was the painting framed, mounted in a square or rectangular frame, or in a round frame (a tondo). Were all the subjects depicted reproduced in the sketches of the participants. Which details in the picture were noted and which were of less importance. This exercise targeted at memorizing the main points (of a patient) and leaving out details that are not relevant to the case.

Seeing through someone else’s eyes

In an exercise, entitled ‘Seeing through someone else’s eyes’, the participants turned their back to a piece of art while one participant was instructed to describe the artwork to the others. Based on the description they received; the participants were asked to draw the picture on a sheet of paper. The sketches were placed side by side and discussed in the group in face of the actual work of art. The disparate interpretations of the transmitted information became apparent in the resulting sketches, showing the differences between observations of the describer and interpretations of the receivers. This is recognized in clinical practice when rendering information regarding patients among colleagues.

Embodiment

In this exercise, named ‘embodiment’, a participant described the posture of a sculptured subject to a partner who was blinded to the sculpture. According to the bodily information, the listener would take the stand and posture represented by the sculpture. Photographs were taken using smartphones and compared with the sculpture. This exercise requires translating someone else’s instructions into one’s own physical perception.

**Evaluation of iconodiagnostics**

Iconodiagnostics added a medical stance in observing the artworks. Most of the paintings or sculptures that were selected, revealed a component that could be associated with a medical condition, according to features depicted by the artists, whether intended or by coincidence. These associations were obviously made on the basis of visual elements in the work of art as recognized from known clinical attributes, as outlined by the senior clinician.
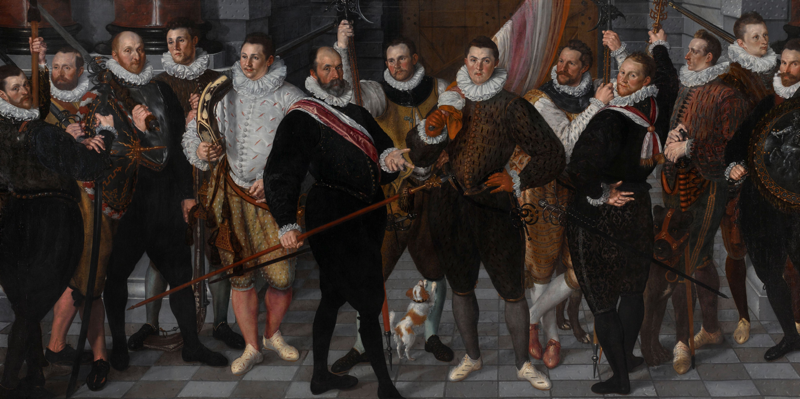


Fig. 3: The Company of Captain Dirck Jacobsz Rosecrans and Lieutenant Pauw, Cornelis Ketel, 1588, oil on canvas, h 208cm × w 410cm, Rijksmuseum Amsterdam*.*
