# Supplementary material for: Empathy and tolerance of ambiguity in medical students and doctors participating in art-based observational training at the Rijksmuseum in Amsterdam, the Netherlands: a before-and-after study
Source: J Educ Eval Health Prof. 2025 Jan 14;22:3. doi: 10.3352/jeehp.2025.22.3 (PMC11880821; doi:10.3352/jeehp.2025.22.3)
Supplement: Supplementary file 4 — Supplement 2. Jefferson Scale of Empathy. [file jeehp-22-03-suppl2.docx]

Supplement 2. Jefferson Scale of Empathy

Instructions: using a ball-point pen, please indicate the extent of your agreement or disagreement with each of the following statements by marking the appropriate circle to the right of each statement.

Please use the following 7-point scale (a higher number on the scale indicates more agreement): mark one and only one response for each statement.

|  | 1 2 3 4 5 6 7 |
| --- | --- |
| 1. My understanding of how my patients and their families feel does not influence medical or surgical treatment | O O O O O O O |
| 1. My patients feel better when I understand their feelings | O O O O O O O |
| 1. It is difficult for me to view things from my patients' perspectives | O O O O O O O |
| 1. I consider understanding my patients' body language as important as verbal communication in caregiver- patient relationships | O O O O O O O |
| 1. I have a good sense of humour that I think contributes to a better clinical outcome | O O O O O O O |
| 1. Because people are different, it is difficult for me to see things from my patients' perspectives | O O O O O O O |
| 1. I try not to pay attention to my patients' emotions in history taking or in asking about their physical health | O O O O O O O |
| 1. Attentiveness to my patients' personal experiences does not influence treatment outcomes | O O O O O O O |
| 1. I try to imagine myself in my patient's shoes when providing care to them | O O O O O O O |
| 1. My patients value my understanding of their feelings which is therapeutic in its own right | O O O O O O O |
| 1. Patients' illnesses can be cured only by medical or surgical treatment, therefore, emotional ties to my patients do not have a significant influence on medical or surgical outcomes | O O O O O O O |
| 1. Asking patients about what is happening in their personal lives is not helpful in understanding their physical complaints | O O O O O O O |
| 1. I try to understand what is going on in my patients' minds by paying attention to their non-verbal cues and body language | O O O O O O O |
| 1. I believe that emotion has no place in the treatment of medical illness | O O O O O O O |
| 1. Empathy is a therapeutic skill without which success in treatment is limited | O O O O O O O |
| 1. An important component of the relationship with my patients is my understanding of their emotional status, as well as that of their families | O O O O O O O |
| 1. I try to think like my patients in order to render better care | O O O O O O O |
| 1. I do not allow myself to be influenced by strong personal bonds between my patients and their family members | O O O O O O O |
| 1. I do not enjoy reading non-medical literature or the arts | O O O O O O O |
| 1. I believe that empathy is an important therapeutic factor in medical or surgical treatment | O O O O O O O |
