# Supplementary material for: Empathy and tolerance of ambiguity in medical students and doctors participating in art-based observational training at the Rijksmuseum in Amsterdam, the Netherlands: a before-and-after study
Source: J Educ Eval Health Prof. 2025 Jan 14;22:3. doi: 10.3352/jeehp.2025.22.3 (PMC11880821; doi:10.3352/jeehp.2025.22.3)
Supplement: Supplementary file 5 — Supplement 3. Tolerance of Ambiguity in Medical Students and Doctors scale. [file jeehp-22-03-suppl3.docx]

Supplement 3. Tolerance of Ambiguity in Medical Students and Doctors scale.

Instructions: using a ball-point pen, please indicate the extent of your agreement or disagreement with each of the following statements by marking the appropriate circle to the right of each statement.

Please use the following 5-point scale (a higher number on the scale indicates more agreement): mark one and only one response for each statement.

|  | 1 2 3 4 5 |
| --- | --- |
| 1. I would enjoy tailoring treatments to individual patient problems. | O O O O O |
| 1. I have a lot of respect for consultants who always come up with a definite answer. | O O O O O |
| 1. I would be comfortable if a clinical teacher set me a vague assignment or task. | O O O O O |
| 1. A good clinical teacher is one who challenges your way of looking at clinical problems. | O O O O O |
| 1. What we are used to is always preferable to what is unfamiliar. | O O O O O |
| 1. I feel uncomfortable when people claim that something is ‘absolutely certain’ in medicine. | O O O O O |
| 1. A doctor who leads an even, regular work life with few surprises, really has a lot to be grateful for. | O O O O O |
| 1. I think in medicine it is important to know exactly what you are talking about at all times. | O O O O O |
| 1. I feel comfortable that in medicine there is often no right or wrong answer. | O O O O O |
| 1. A patient with multiple diseases would make a doctor’s job more interesting. | O O O O O |
| 1. I am uncomfortable that a lack of medical knowledge about some diseases means we can’t help some patients. | O O O O O |
| 1. The unpredictability of a patient’s response to medication would bring welcome complexity to a doctor’s role. | O O O O O |
| 1. It is important to appear knowledgeable to patients at all times. | O O O O O |
| 1. Being confronted with contradictory evidence in clinical practice makes me feel uncomfortable. | O O O O O |
| 1. I like the mystery that there are some things in medicine we’ll never know. | O O O O O |
| 1. Variation between individual patients is a frustrating aspect of medicine. | O O O O O |
| 1. I find it frustrating when I can’t find the answer to a clinical question. | O O O O O |
| 1. I am apprehensive when faced with a new clinical situation or problem. | O O O O O |
| 1. I feel uncomfortable knowing that many of our most important clinical decisions are based upon insufficient information. | O O O O O |
| 1. No matter how complicated the situation, a good doctor will be able to arrive at yes or no answer. | O O O O O |
| 1. I feel uncomfortable when textbooks or experts are factually incorrect. | O O O O O |
| 1. There is really no such thing as a clinical problem that can’t be solved. | O O O O O |
| 1. I like the challenge of being thrown in the deep end with different medical situations. | O O O O O |
| 1. It is more interesting to tackle a complicated clinical problem than to solve a simple one. | O O O O O |
| 1. I enjoy the process of working with a complex clinical problem and making it more manageable. | O O O O O |
| 1. A good job is one where what is to be done and how it is to be done are always clear. | O O O O O |
| 1. To me, medicine is black and white. | O O O O O |
| 1. The beauty of medicine is that it’s always evolving and changing. | O O O O O |
| 1. I would be comfortable to acknowledge the limits of my medical knowledge to patients. | O O O O O |

*Scoring*

*If you wish to compare your scores to our published study, you will need to calculate your TAMSAD score out of 100 using the following steps:*

- *Step 1 Reverse the codes for the items asterisked (e.g. a 2 becomes a 4).*
- *Step 2 Calculate your mean score out of 5 across the 29 items (e.g. 3.14).*
- *Step 3 Transform your mean score from a 1–5 scale to a 0–100 scale using the formula: New score = 25 (Old score - 1). So, for example, using the previous example, the new score would be 25(3.14-1) = 25*2.14 = 53.5.*
