# Supplementary material for: Empathy and tolerance of ambiguity in medical students and doctors participating in art-based observational training at the Rijksmuseum in Amsterdam, the Netherlands: a before-and-after study
Source: J Educ Eval Health Prof. 2025 Jan 14;22:3. doi: 10.3352/jeehp.2025.22.3 (PMC11880821; doi:10.3352/jeehp.2025.22.3)
Supplement: Supplementary file 6 — Supplement 4. Qualitative evaluation form with open-ended responses. [file jeehp-22-03-suppl4.docx]

Supplement 4. Qualitative evaluation form with open-ended responses.

Instructions: Please indicate the extent of your agreement or disagreement with each of the following statements by marking the appropriate circle to the right of each statement.

Please use the 5-point scale (a higher number on the scale indicates more agreement): mark one and only one response for each statement. For every question there is the possibility to expand on the answer

|  | 1 2 3 4 5 |
| --- | --- |
| 1. I am interested in art in everyday life. | O O O O O |
|  |  |
| 1. I found this course useful. | O O O O O |
|  |  |
| 1. I think this course could promote delaying my judgement of a situation (until all information has been gathered). | O O O O O |
|  |  |
| 1. This course contributes to raising awareness of my own judgement and the prejudices I carry. | O O O O O |
|  |  |
| 1. The above elements contribute to my professional development as a doctor. | O O O O O |
|  |  |
| 1. This course improves my ability to empathize with a patient. | O O O O O |
|  |  |
| 1. This course promotes physician-physician communication. | O O O O O |
|  |  |
| 1. This course contributes to the promotion of team building. | O O O O O |
|  |  |
| 1. The addition of a medical aspect to the artwork is useful. | O O O O O |
|  |  |
